# The seroepidemiology of the chronic infections in patients with myocardial infarction in North of Iran

**Published:** 2010

**Authors:** Hadi Bazzazi, Ezzat Allah Ghaemi, Mohammad Ali Ramezani

**Affiliations:** a Department of Laboratory Sciences, Islamic Azad University, Gorgan Branch, Gorgan, Iran; b Associate Professor, Microbiology Department, Golestan University of Medical Sciences, Gorgan, Iran; c Associate Professor, Heart Department, Golestan University of Medical Sciences, Gorgan, Iran

**Keywords:** Myocardial Infarction, Helicobacter Pylori, Chlamydiae Pneumoniae, IgA, IgG

## Abstract

**BACKGROUND:**

Recent studies have suggested that chronic infections with Chlamydia pneumoniae (Cpn) and Helicobacter pylori (Hp) may be associated with the risk of Myocardial Infarction (MI).

**METHODS:**

A cross sectional study was conducted on 140 citizens. Seroprevalence was assessed by ELISA tests measuring IgA and IgG antibodies to Cpn and Hp in sera.

**RESULTS:**

Among patients, %11.4 and %90.0 were seropositive for Anti-Cpn IgA and IgG respectively, and also %51.4 and %58.6 were seropositive for Anti-Hp IgA and IgG respectively.

**CONCLUSIONS:**

The present study shows that previous infection to Cpn in patients with MI is important. But there are no significant association between infection with Hp and MI.

Current studies have suggested that chronic infections, especially those that caused by Chlamydia pneumoniae (Cpn) and Helicobacter pylori (Hp) may be linked with the risk of Myocardial Infarction (MI).^1^,^2^ Since 1988 that Saikku et al demonstrated that patients with recent myocardial infarction had significantly elevated IgG and IgA antibody titers against Cpn compared with normal controls, several seroepidemiological studies from different regions have been published, with conflicting results.^3^–^6^ Additionally, following the initial report by Mendall et al in 1994, several seroepidemiological studies have been carried out on the association between Hp infection and coronary heart disease (CHD).^7^,^8^ Some researchers suggested that acute myocardial infarction might be associated with an acute exacerbation of chronic infection. The mechanism by which chronic infections influence the progress of CHD is not known. Determining risk factors of the MI is of huge significance, so this comparative study was carried out to determine antibody level against Cpn and Hp in patient with MI and healthy subjects.

## Methods

A case-control, cross sectional study was conducted on 140 subjects. The study was carried out in 2006 in the city of Gorgan, the center of Golestan province, located in the Southeast of the Caspian Sea. The two groups were similar regarding age. Cases (n = 70, mean age of 54.1 years) defined as patients who presented clinically as myocardial infarction (MI). MI was diagnosed as continuous ischemic chest pain within 24 hours of presentation, rise of creatine kinase to twice the upper limit of normal for at least two times and characteristic electrocardiography changes in the ST segment. Those who had no definite typical electrocardiography changes were served as controls (n = 70, mean age of 53.7 years). IgG and IgA antibodies against Cpn and Hp were tested using ELISA according to manufacturer’s instructions. The results were determined by calculating an index value from optical density values relative to control materials. Seropositivity was defined as the presence of either IgG or IgA antibodies. Collected data were analyzed with the Epi-Info statistical software (CDC-USA) and statistical significant difference was assessed by Fisher exact test and Chi-square test. P value < 0.05 was considered significant.

## Results

Each group (Patients and Controls) consisted of 22 (39.4%) female and 48 (68.6%) male samples. Age and sex difference between patients and the controls was not statistically significant (p > 0.05). Table 1 shows the frequency of anti-Cpn and Anti-Hp antibodies in serum of patient and control groups. Table 2 shows the frequency of anti-Chlamydia pneumoniae IgG and IgA isotypes in serum of patient and control groups. Table 2 also shows the frequency of anti-Helicobacter pylori IgG and IgA iso-types in serum of patient and control groups.

**Table 1:** Frequency of anti-Cpn and Anti-Hp antibodies in serum of patient and control groups

	Study Group		
Antibody	Patient n (%)	Control n (%)	P value
Anti-Cpn IgG	63 (%90.0)	54 (%77.1)	< 0.02
Anti-Cpn IgA	8 (%11.4)	14 (%20.0)	> 0.05
Anti-Hp IgG	41 (%58.6)	41 (%58.6)	> 0.05
Anti-Hp IgA	36 (%51.4)	24 (%34.3)	< 0.02

**Table 2:** Frequency of IgG and IgA isotypes of anti-Chlamydia pneumoniae and anti-Helicobacter pylori in serum of patient and control groups

IgG in serum	IgA in serum	Anti-Cpn percent in control (n)	Anti-Cpn percent in patient (n)	Anti-Hp percent in control (n)	Anti-Hp percent in patient (n)
+	+	18.6% (13)	11.4% (8)	20.0% (14)	41.4% (29) ***
+	-	58.6% (41)	78.6% (55) *	38.6% (27)	17.1% (12) ****
-	+	1.4% (1)	0.0% (0)	14.3% (10)	10.0% (7)
-	-	21.4% (15)	10.0% (7) **	27.1% (19)	31.4% (22)

* < 0.005

** < 0.03

*** < 0.003

**** < 0.002

## Discussion

According to the results of this study, the high prevalence of IgG in cases and in controls demonstrates that prevalence of previous infection with Cpn in our region is high. A possible reason for the high prevalence may be that the study population was old. In this study, an association between IgG isotype to Cpn and prevalence of MI was observed. This fact suggests that previous infection to Cpn in patients with MI is important. This result is in agreement with a number of seroepidemiological studies.^9^,^10^ Additionally, as shown in table 2, occurrence of IgG without IgA in serum, have case-control significance differences. Existence of IgG in the absence of IgA to Cpn may be considered as a sign of past infection while in reinfection the IgA response is predominant.^11^ Prevalence of IgA class of Anti-Cpn in cases and in controls shows that acute infection is not infrequent in this area as well. On the other hand, frequency in control population was significantly greater than cases, in condition that nothing of IgG and IgA to Cpn in sera is detected. It is indicated that in people who have not an evidence of infection with Cpn, possibility of the MI incidence would be reduced. The studies of relation between Cpn and heart disease have created diverse results in spite of whether IgG or IgA was measured.^12^–^14^ The basis for the inconsistency is unidentified, but various explanations have been suggested incl udes differences in techniques, titer limits, study populations, and the used sampling time.

On the basis of serpositivity to entire Anti-Hp IgG and IgA isotypes, population can be separated to infect with Hp and healthy groups. As indicated in table 2, %68.57 of patients and %69.86 of controls are seropositive to total anti-Hp specific IgA and IgG isotypes and so it seems that frequency of infection in two groups is comparable. Furthermore, as shown in table 1, there was no important difference in frequency of whole anti-Hp IgG antibodies between patients and controls. The present findings agree with the results of some of the previous studies that found no significant association between infection with Hp as evidenced by elevated antibodies to Hp and acute myocardial infarction.^15^,^16^ But a number of other studies have reported more prevalence of anti-Hp antibodies in serum of patient with ACS in comparing to controls.^17^

## Conclusions

The present findings show that previous infection to Cpn in patients with MI is important. But there are no significant association between infection with Hp as evidenced by elevated antibodies to Hp and MI.

## References

[1] Roivainen M, Viik-Kajander M, Palosuo T, Toivanen P, Leinonen M, Saikku P (2000). Infections, inflammation, and the risk of coronary heart disease. Circulation.

[2] Lamb DJ, Ferns GAA (1999). Infection, immunisation and atherosclerosis: is there a link?. Vaccine.

[3] Saikku P, Leinonen M, Mattila K, Ekman MR, Nieminen MS, MȨkelȨ PH (1988). Serological evidence of an association of a novel Chlamydia, TWAR, with chronic coronary heart disease and acute myocardial infarction. Lancet.

[4] Grayston JT (2000). Background and current knowledge of Chlamydia pneumoniae and atherosclerosis. J Infect Dis.

[5] Mendall MA, Carrington D, Strachan D, Patel P, Molineaux N, Levi J (1995). Chlamydia pneumoniae: risk factors for seropositivity and association with coronary heart disease. J Infect.

[6] Saikku P (2000). Epidemiologic association of Chlamydia pneumoniae and atherosclerosis: the initial serologic observation and more. J Infect Dis.

[7] Mendall MA, Goggin PM, Molineaux N, Levy J, Toosy T, Strachan D (1994). Relation of helicobacter pylori infection and coronary heart disease. Br Heart J.

[8] Danesh J, Youngman L, Clark S, Parish S, Peto R, Collins R (1999). Helicobacter pylori infection and early onset myocar-dial infarction: case-control and sibling pairs study. BMJ.

[9] Chandra HR, Choudhary N, O’Neill C, Boura J, Timmis GC, O’Neill WW (2001). Chlamydia pneumoniae exposure and inflammatory markers in acute coronary syndrome (CIMACS). Am J Cardiol.

[10] Espinola-Klein C, Rupprecht HJ, Blankenberg S, Bickel C, Kopp H, Rippin G (2000). Are morphological or functional changes in the carotid artery wall associated with Chlamydia pneumoniae, helicobacter pylori, cytomegalovirus, or herpes simplex virus infection?. Stroke.

[11] von Hertzen L, Leinonen M, Surcel HM, Karajlainen J, Saikku P (1995). Measurement of sputum antibodies in the diagnosis of acute and chronic respiratory infections associated with Chlamydia pneumonia. Clin Diagn Lab Immunol.

[12] Strachan DP, Carrington D, Mendall MA, Ballam L, Morris J, Butland BK (1999). Relation of Chlamydia pneumoniae serology to mortality and incidence of ischaemic heart disease over 13 years in the caerphilly prospective heart disease study. BMJ.

[13] Kosaka C, Hara K, Komiyama Y, Takahashi H (2000). Possible role of chronic infection with Chlamydia pneumoniae in Japanese patients with acute myocardial infarction. Jpn Circ J.

[14] Mendis S, Arseculeratne YM, Withana N, Samitha S (2001). Chlamydia pneumoniae infection and its association with coronary heart disease and cardiovascular risk factors in a sample South Asian population. Int J Cardiol.

[15] De Backer J, Mak R, De Bacquer D, Van Renterghem L, Verbraekel E, Kornitzer M (2002). Parameters of inflammation and infection in a community based case-control study of coronary heart disease. Atherosclerosis.

[16] Franceschi F, Leo D, Fini L, Santoliquido A, Flore R, Tondi P (2005). Helicobacter pylori infection and ischaemic heart disease: an overview of the general literature. Dig Liver Dis.

[17] Miyazakia M, Babazono A, Kadowaki K, Kato M, Takata T, Une H (2006). Is helicobacter pylori infection a risk factor for acute coronary syndromes?. J Infect.

